# Implementing a Standardized Nurse-driven Rounding Protocol in a Trauma-surgical Intensive Care Unit: A Single Institution Experience

**DOI:** 10.7759/cureus.3422

**Published:** 2018-10-08

**Authors:** Clement D Marshall, Maureen E Fay, Brian Phillips, Robert Faurote, Jamie Kustudia, Ryan C Ransom, Christine Henley, Lisa DiConstanzo, Jeffrey K Jopling, Adam X Sang, David A Spain, Julie A Tisnado, Thomas G Weiser

**Affiliations:** 1 Surgery, Stanford Hospital, Stanford, USA; 2 Critical Care, Stanford Hospital, Stanford, USA

**Keywords:** communication, critical care, team work, sicu

## Abstract

Introduction

Patient care in the trauma-surgical intensive care unit (SICU) requires trust and effective communication between nurses and physicians. Our SICU suffered from poor communication and trust between nurses and physicians, negatively impacting the working environment and, potentially, patient care.

Methods

A SICU Task Force studied communication practices and identified areas for improvement, leading to several interventions. The daily physician rounding was altered to improve communication and to enhance the role of the registered nurses (RN) in rounds. Additionally, a formal night resident rounding time was implemented.

Results

A post-intervention survey focusing on cooperation, teamwork, and appreciation between nurses and physicians revealed improvement in these domains. Informal feedback from nurses and physicians indicated improved working relationships and satisfaction with the SICU environment. However, results of a national survey performed after the intervention did not show the same level of improvement.

Conclusions

A Task Force consisting of SICU nurses and physicians can effectively study a widespread communication issue and implement targeted interventions. While informal feedback may indicate improvement, it can be difficult to demonstrate improvement using formal surveys.

## Introduction

A trauma hospital in which registered nurses (RNs) and physicians (MDs) engage in effective communication and teamwork is the optimal environment for promoting patient healing and recovery [1, 2]. Conversely, poor communication between nurses and physicians (RN-MD) has been identified as a leading cause of adverse events [3, 4]. Critically ill trauma patients are usually cohorted in trauma-surgical intensive care units (SICUs), where they are cared for by multiple different health care professionals around the clock. The task-dense and hectic environment of the SICU makes it easy for poor communication patterns to persist [5, 6]. Morning rounding has been identified as a valuable target for improving communication [3, 7], so we wished to study whether implementing a nurse-driven daily rounding format in the SICU can improve the quality of RN-MD communication.

## Materials and methods

**Figure 1:**
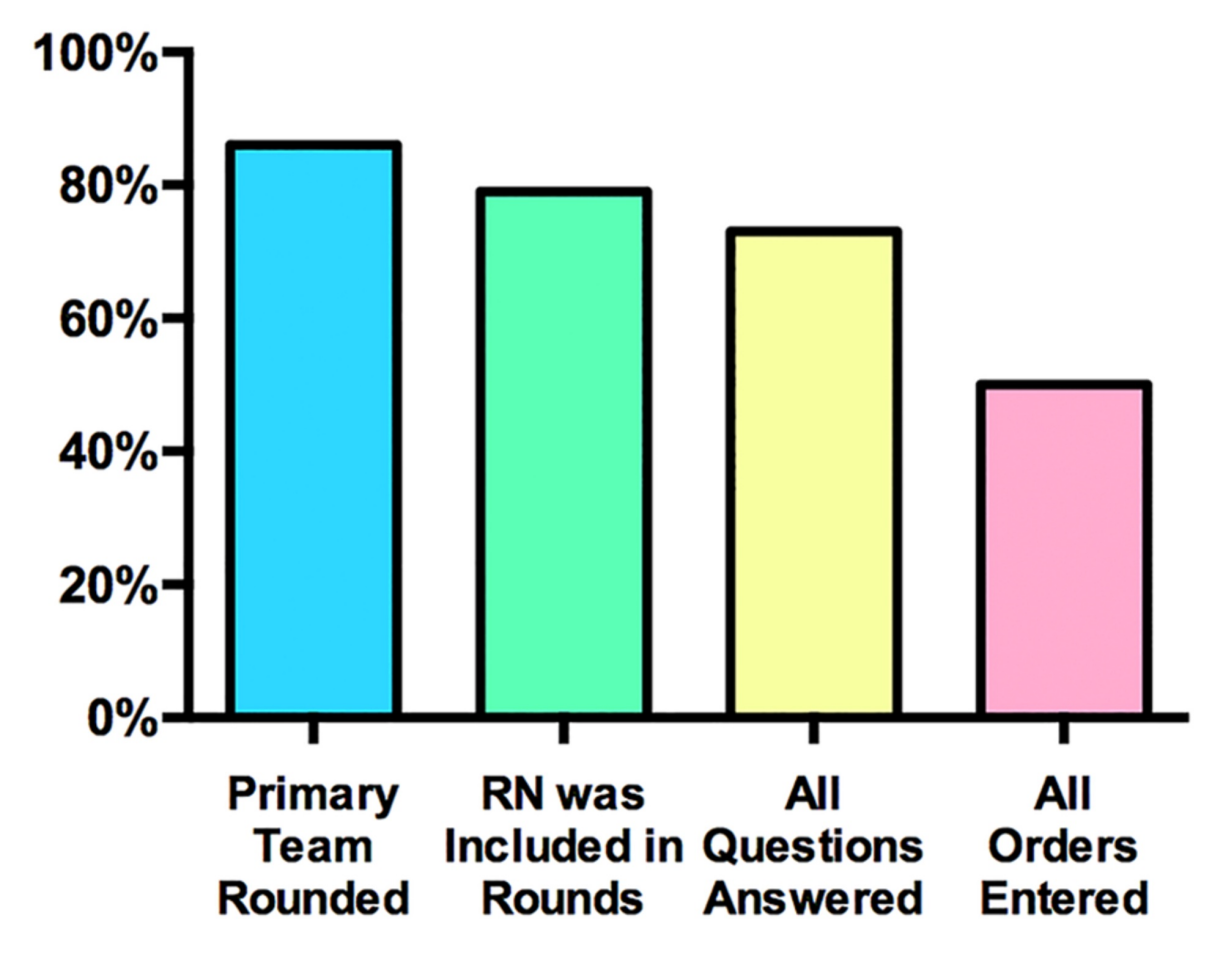
Survey of SICU morning rounding practices prior to the intervention Results indicated room for improvement, particularly with the “Questions answered” and “Orders entered” metrics. RN, nurse.

The setting for this study is a SICU in a quaternary academic medical center. RNs in this unit care for SICU and medical ICU (MICU) patients but the study was confined to the SICU physician team and patients. Prior to the study period, the prevailing perception in the SICU was that communication and teamwork between physicians and nurses was suboptimal. Results from SICU nurses of the 2014 National Database of Nursing Quality Indicators (NDNQI) Survey indicated lower than average perception of the quality of RN-MD interaction, cooperation, teamwork, and appreciation. In response to these survey results, a Task Force consisting of bedside SICU RNs, SICU nursing leadership, SICU attending MDs, and surgical residents was created. The Task Force identified morning rounds as the optimal opportunity for intervention. Four aspects of morning rounding were chosen for further study: (1) whether the primary MD team rounded with the RN present, (2) whether the RN was included in the rounds, (3) whether all questions from the RN were answered by the primary team, and (4) whether all orders were entered by the end of the rounds. To measure the baseline level of adherence to these measures, SICU RNs were asked to answer a daily survey consisting of the same four questions. Every day over a one month period, RNs for each SICU patient completed a survey on the quality of MD team rounding (Range 5-11 patients per day, average eight per day. 100% survey completion rate.) The results of the survey indicated substantial room for improvement with these measures (Figure 1).

Based on these results, the Task Force chose three interventions to improve communication during morning rounding: (1) standardize the rounding process, (2) expand and standardize the nurse’s role in morning rounds, and (3) institute a nightly check-in between the overnight nurse and the resident. In addition, the Task Force designated SICU RN Champions to advocate for and implement these changes.

Standardization of the MD rounding process

**Figure 2:**
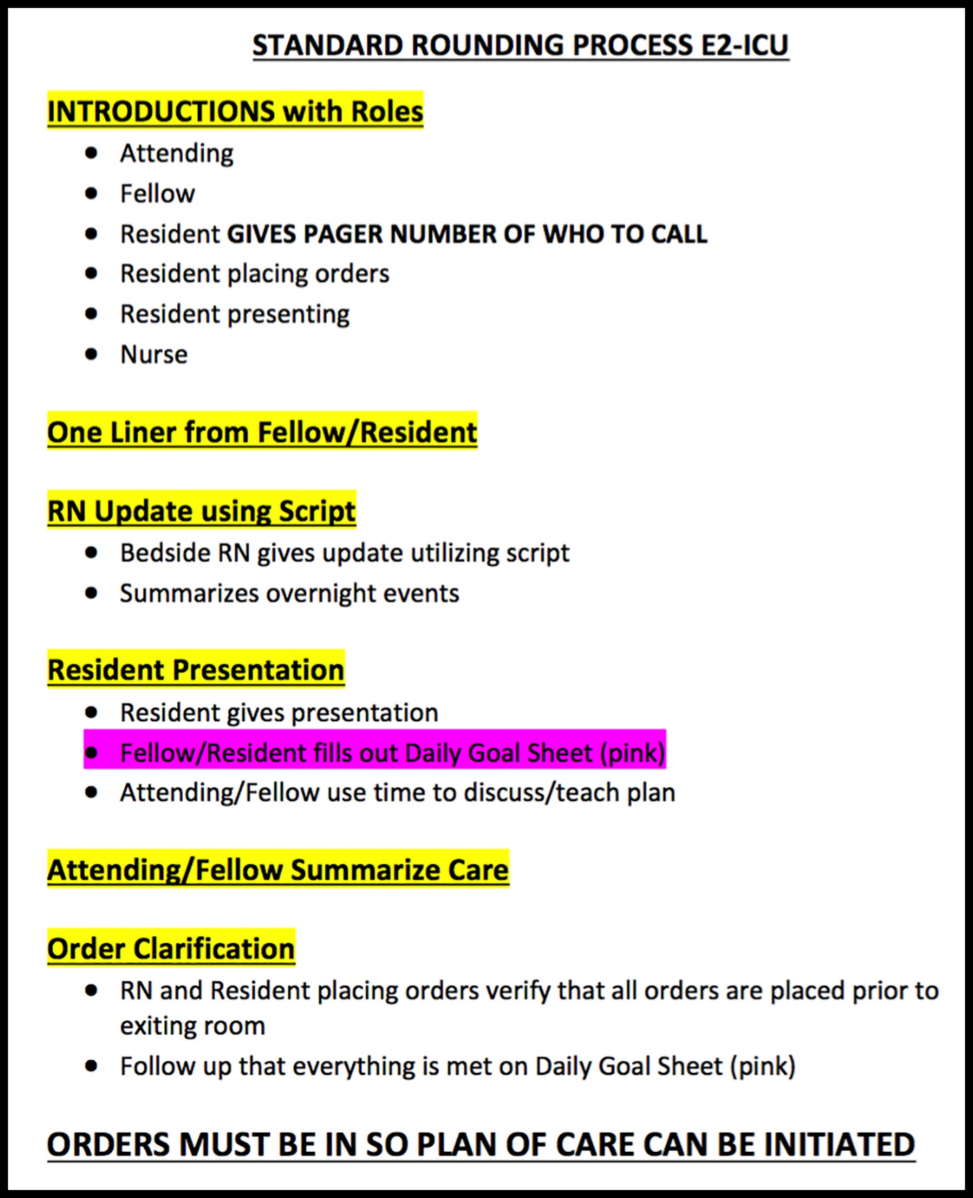
The standardized SICU morning rounding script The important elements being introduced are the Introductions, the RN presentation, and the order clarification. E2-ICU, E2 Intensive Care Unit. RN, nurse.

Informal surveys of SICU RNs indicated that elements of morning rounding that could benefit from standardization included identification of key MD team members (the SICU attending, the chief resident or fellow, and the primary resident responsible for managing the patient) and confirmation of order entry by the end of rounding. To improve these aspects of rounding, a standardized script for rounding was developed (Figure 2).

For each patient:

1.     Several roles are established: (1) the resident presenting the patient, (2) a resident to enter orders in real time during rounds using a mobile computer workstation, and (3) a resident to triage phone calls and pages during rounding.

2.     For each patient, the bedside RN and key members of the MD team introduce themselves, following which the RN delivers a structured presentation containing key updates and concerns (discussed in more detail below).

3.     Following the RN presentation, the primary resident delivers a traditional systems-based presentation on the patient’s status, followed by a team discussion and development of the plan for the day.

4.     New orders are entered in real time by the order entry resident and at the end of the discussion, this resident reads back all new orders that have been entered or modified. Rounding ends when all team members agree that all questions and concerns have been addressed and that all relevant orders have been entered.

To demonstrate an idealized version of the new rounding process, an instructional video was produced by the Task Force and was integrated into in-service training for SICU RNs and MD teams (Video 1). 

**Video 1:** Example SICU rounding video A video was created to demonstrate the “ideal’ rounding process including all of the Task Force’s interventions.

Standardization of RN participation in rounds

**Figure 3:**
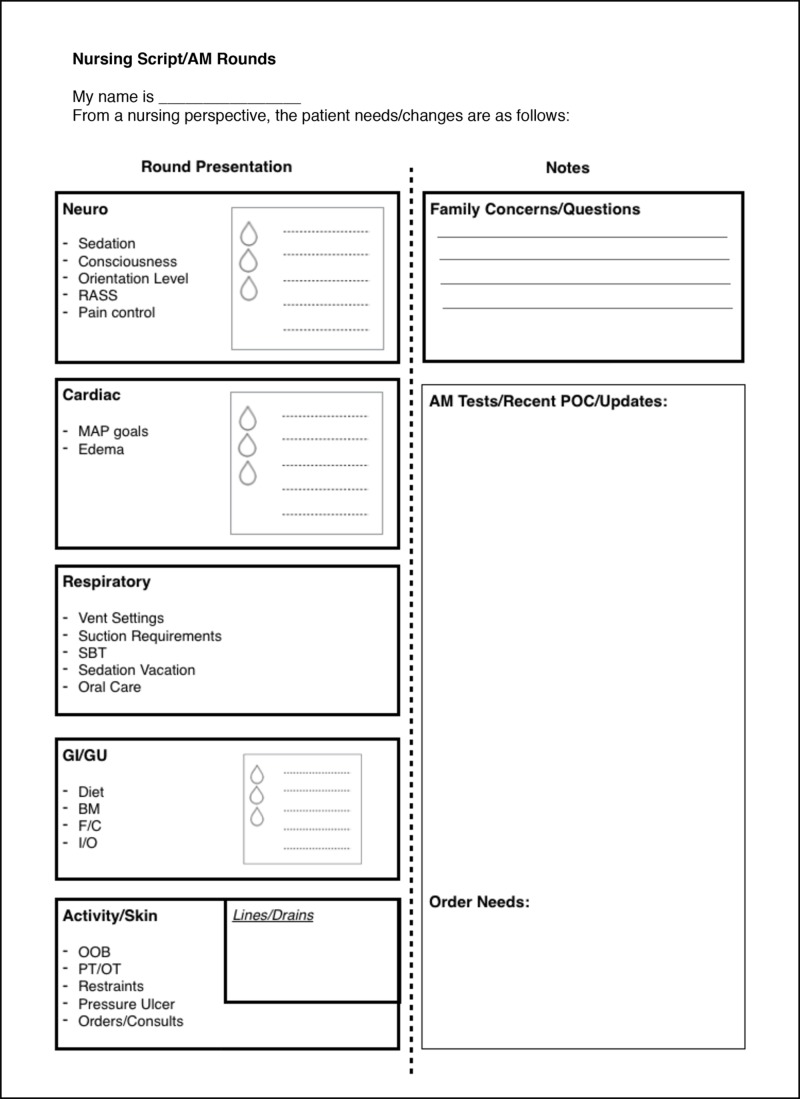
Rounding script This script serves as a template for the RN presentation during morning rounding. RASS, Richmond Agitation-Sedation Scale. MAP, mean arterial pressure. SBT, spontaneous breathing trial. BM, bowel movement. F/C, Foley catheter. I/O, ins and outs. OOB, out of bed. PT/OT, physical therapy, occupational therapy. POC, plan of care.

A common concern among SICU RNs was that there was not a consistent and integral role for the bedside RN during morning rounding. While some felt comfortable taking an active role in rounds discussions, others felt that their voice was not always heard and that their concerns or questions were not consistently addressed. To improve this situation, a standardized, structured RN presentation was integrated into the rounding workflow. The RN presentation was summarized into a sheet to be used by the RN during rounds (Figure 3).

The presentation consists of updates on the patient’s status with regard to several systems: (1) neurological, including level of consciousness, pain control, and rates of sedative and analgesic medications, (2) cardiac, including rates of cardiac and vasoactive drips, (3) respiratory, including current vent settings, suctioning requirements, and spontaneous breathing trial results, (4) gastrointestinal and genitourinary, including current diet or feeding rate, and recent fluid balance, (5) activity and skin, including out-of-bed time, need for restraints, and skin or ulcer concerns, and (6) family questions or concerns as well as any RN concerns. The RN presentation was designed to give the most up-to-date clinical information, as well as to avoid the frequent situation in which the RN must correct the presenting resident with more current information, such as updated ventilator settings or drip rates.

Nightly check-in

**Figure 4:**
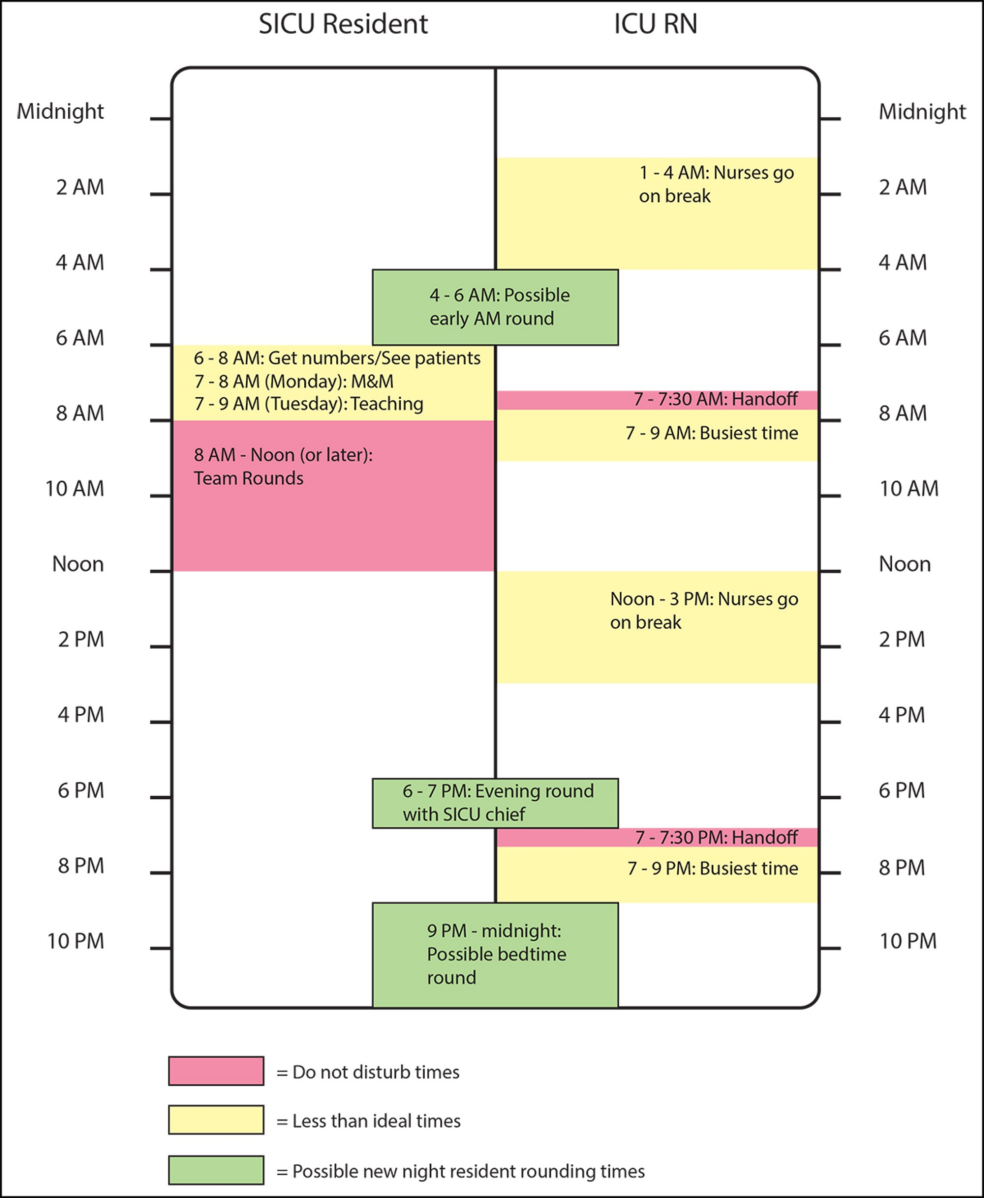
Weekday workflow Summary of the typical weekday workflow for SICU RNs and MDs that was used to identify an ideal time for the nightly check-in. Both 9 PM to midnight and 4 to 6 AM “overnight rounding” times are given as options, depending on degree of activity of the night shift. M&M, mortality and morbidity conference. SICU, surgical intensive care unit.

Many overnight RNs felt that more frequent and regular communication with the overnight resident would prevent non-urgent issues from accumulating and needing to be addressed on morning rounds and would generally improve rapport between the resident and RNs. To address this concern, a standardized nightly check-in was instituted. Based on an examination of the daily work flow for SICU residents and RNs (Figure 4), two check-in periods (one from 9 PM to midnight and one from 4 to 6 AM) were chosen. During these times, the overnight resident is expected to visit, in-person, each patient and the patient’s bedside nurse in order to address any issues or place any orders requested by the RN. It was recognized that on some nights emergencies might prevent the check-in from happening with each patient, but the Task Force believed that instituting a formal expectation would help to improve both workflow and RN-MD communication.

Designation of SICU RN Champions

**Figure 5:**
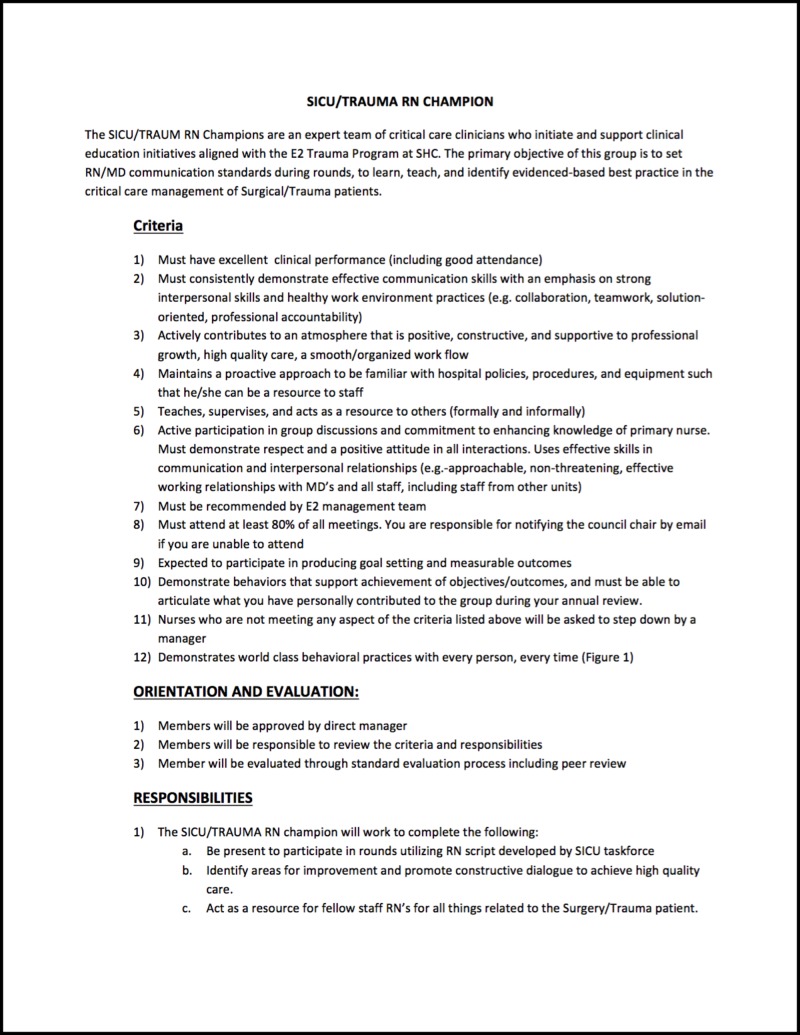
Criteria and responsibilities for the SICU RN Champions SICU, surgical intensive care unit. SHC, Stanford Hospital Center. RN/MD, nurse/physician.

In order to improve integration of the above interventions into the SICU workflow and to facilitate the flow of feedback, a SICU RN Champion program was initiated. A group of SICU RN volunteers, several of whom were also members of the Task Force, were designated as SICU RN Champions (Figure 5).

These RNs were briefed on the new rounding work flow and the RN script, as well as the night resident check-in. Over the course of several weeks the SICU Champions provided feedback to the Task Force, allowing the implementation of the interventions to adapt in real time. Prior to implementing the interventions and during the implementation period, the SICU Champions also helped to educate other RNs caring for SICU patients on the new interventions.

## Results

Implementation of the interventions

The standardized MD rounding process was implemented in the SICU daily workflow by several methods. First, the SICU attendings directly encouraged use of the new process by modeling behavior and by reiterating the importance of the process to the residents. Second, the instructional rounding video was shown to all SICU RNs and MDs. Third, eight RN volunteers were chosen to be SICU Champions, and these RNs received instruction on the interventions and watched the rounding instructional video. The SICU Champions then piloted the use of the RN presentation script, which was gradually adopted by all ICU RNs. The nightly check-in was implemented by encouraging the night shift residents to visit and communicate with the RN for each patient during the two overnight windows. Reminders to perform the nightly check-in were given to the night shift residents by SICU attending MDs over the course of the first several weeks of implementation.

Rounding bundle compliance

**Figure 6:**
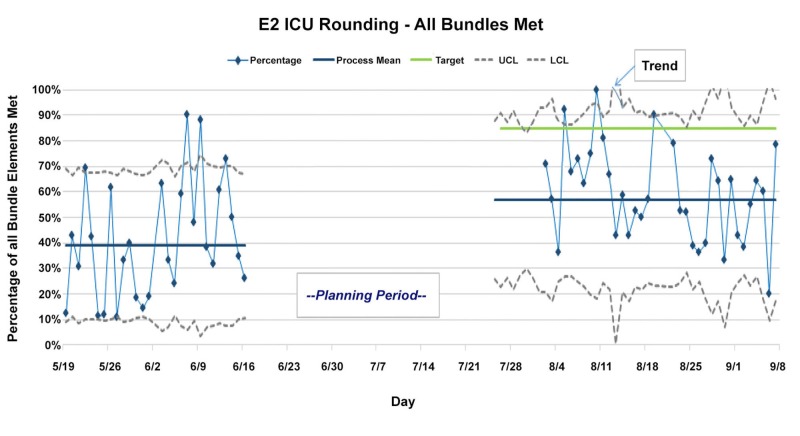
Measurement of compliance with the rounding bundle before and after the intervention period revealed improvement. The bundle being measured consisted of team introductions, RN presentation, and complete order placement E2 ICU, E2 intensive care unit. UCL, upper confidence limit. LCL, lower confidence limit.

To measure improvement in daily rounding, we defined a rounding “bundle” to include the following key questions: (1) Did key team members introduce themselves? (2) Did the bedside RN perform the new RN presentation? (3) Were all orders placed before the MD team moved on to the next patient? For one month prior to the implementation of the improved rounding process, a survey was done to measure baseline compliance with the rounding bundle. The same survey was done over a month after the new measures were implemented. A comparison of compliance before and after the implementation reveals substantial improvement in average compliance with the rounding bundle (Figure 6).

Night check-in compliance

**Figure 7:**
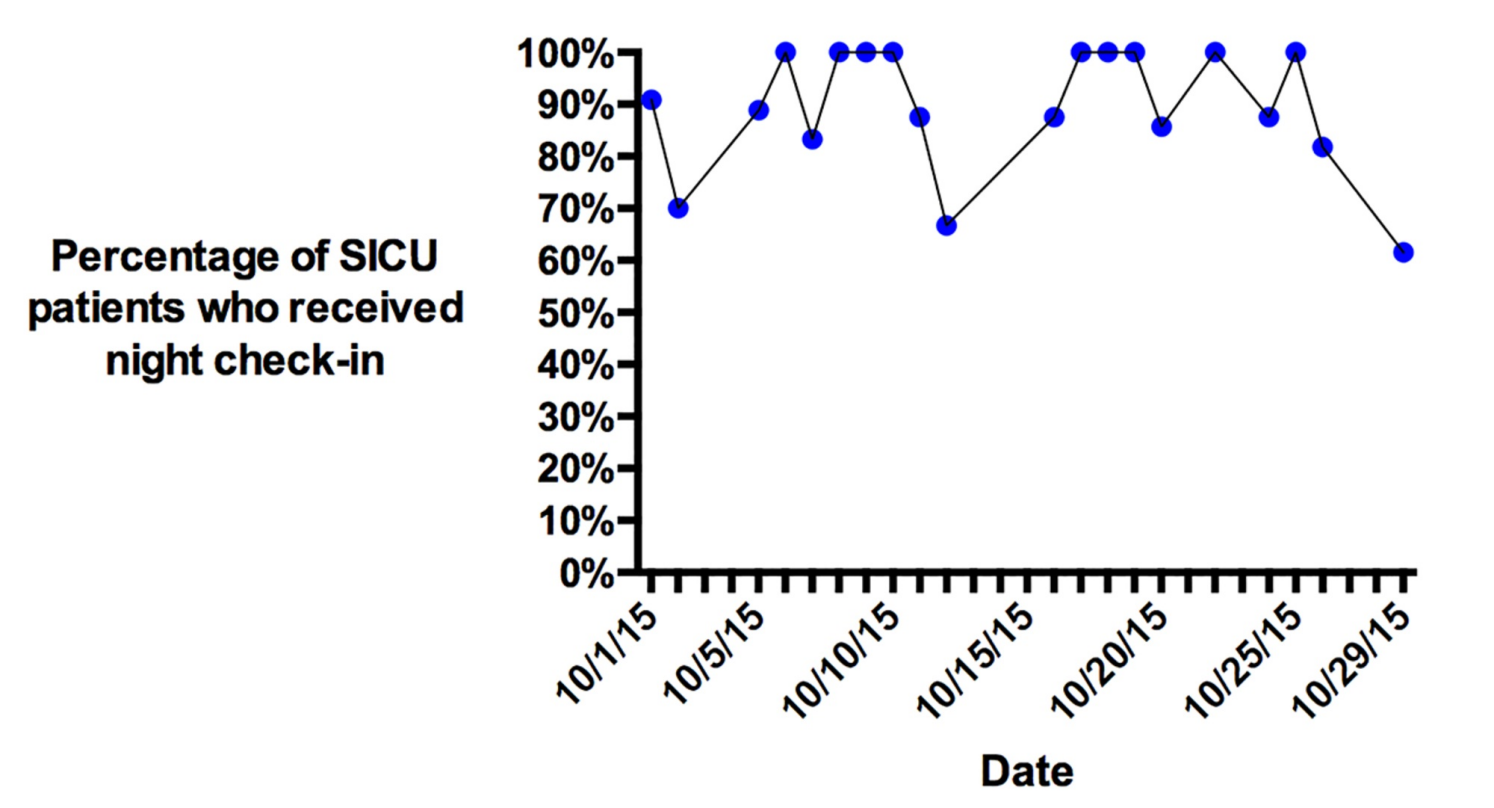
Results of the one-month survey measuring nightly check-in compliance for SICU patients SICU, surgical intensive care unit.

Surveys of night shift RNs were done on a daily basis for a month before and after the implementation. The survey asked whether the overnight resident did an in-person check-in with that nurse during the night shift. Survey results indicated high rates of night check-ins following the implementation (Figure 7).

Survey results

**Figure 8:**
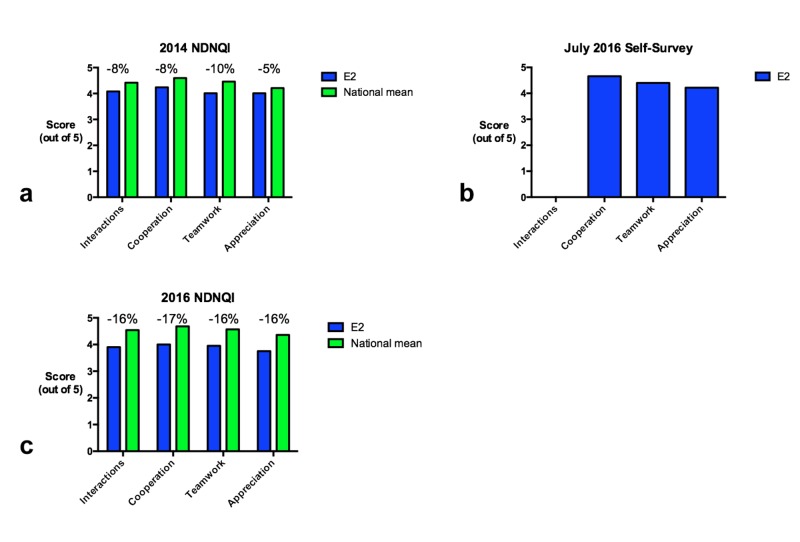
Survey results Results of the 2014 NDNQI (a), a 2016 internal self-survey (b), and the 2016 NDNQI (c). Percentages above bars in the 2014 and 2016 NDNQI survey represent the difference between E2 ICU scores and the national mean score. This comparison was not available for the July 2016 self-survey. Additionally, the Interaction dimension was not assessed in the self survey. E2 indicates our unit, the E2 intensive care unit. NDNQI, national database of nursing quality indicators. ICU, intensive care unit.

NDNQI survey results from September 2014 indicating suboptimal RN perception of RN-MD teamwork within the ICU were a primary motivator for this project. Specifically, the ICU received lower than average scores for questions related to RN-MD interactions, RN-MD cooperation, RN-MD teamwork, and MD appreciation of RN work compared with the national mean (Figure 8a). We therefore wished to use subsequent survey results to assess the effect of implementing the new standardized rounding on RN perception of RN-MD interactions within the ICU. An internal survey containing identical questions to the NDNQI was distributed to ICU RNs in July 2016 and the results indicated substantial improvement in RN perception of RN-MD interactions (Figure 8b). In addition, informal feedback from many ICU nurses and residents indicated that communication and working relationships within the ICU improved substantially since implementation of the improved rounding process. However, the subsequent NDNQI done in September 2016 did not confirm these improvements and in fact revealed slightly lower scores compared with the national mean (Figure 8c).

## Discussion

It is well established that effective and open communication between RNs and MDs is crucial for optimal care of patients in the ICU setting. This is especially true for critically ill trauma patients, who often require complex care involving multiple organ systems. In response to evidence from the 2014 NDNQI survey that RN-MD communication in our ICU was suboptimal, a Task Force was created that studied the problem systematically and implemented several interventions based on input from ICU RNs and MDs. These interventions aimed at standardizing the morning rounding process in a way that improved communication and at improving the participation of the RN in the rounding process. Surveys done before and after the implementation showed improvement in direct measurement of these factors, and an internal survey suggested greater RN satisfaction with their relationship with MDs. However, results from the 2016 NDNQI survey did not demonstrate quantitative improvement in RN perception of RN-MD communication.

There are several possible explanations for this discrepancy. One possible explanation is that during this period there was a high turnover rate in ICU RNs and, as a result, only 65% of the RNs who completed the 2016 survey had also completed the 2014 survey. The remaining 35% of RNs may not have been present for the entire intervention period and may not have had a sense of the improvement that occurred between the two surveys. Another possible explanation is that responses to the NDNQI questions may be indicative of the larger hospital culture, and it is difficult to change culture rapidly. It is possible that, following a successful intervention, it may take longer than two years to observe a meaningful change in culture that would be reflected in improved NDNQI survey results. Furthermore, the ICU environment at our facility is a mixed medical-trauma-surgical unit; thus only a portion of patients and rounding teams were actually involved in the process, while the NDNQI surveyed all ICU nurses. However, based on the results of our internal survey and informal feedback from ICU RNs, we believe that our intervention has improved communication between the RNs and MDs caring for trauma patients in this ICU.

## Conclusions

We instituted several interventions in our trauma and surgical ICU aimed at improving communication and teamwork between RNs and MDs. Informal feedback indicated greater satisfaction among RNs and MDs with the working environment. However, these results were not reproduced on a subsequent national survey, reflecting the difficulty of measuring intervention effectiveness using standardized surveys. The fundamental elements of our approach that may be used in future interventions include: (1) accurately defining the problem, (2) choosing an appropriate setting for an intervention, (3) getting feedback from all stakeholders, (4) implementing a limited number of carefully chosen interventions, and (5) measuring the effectiveness of the interventions. We believe that this process can be a model for nursing units in other hospitals that wish to make improvements.
